# Robot-assisted cannulated compression screw internal fixation for treatment of femoral neck fracture in children: A case series of ten patients

**DOI:** 10.3389/fped.2022.1105717

**Published:** 2023-01-09

**Authors:** Wei Feng, Ziming Yao, Haonan Liu, Dong Guo, Danjiang Zhu, Baojian Song, Qiang Wang, Xuejun Zhang

**Affiliations:** Department of Orthopedics, Beijing Children’s Hospital, Capital Medical University, National Center for Children’s Health, Beijing, China

**Keywords:** robot-assisted, internal fixation, femoral neck fracture, children, screw

## Abstract

**Purpose:**

To investigate the safety and efficacy of robot-assisted cannulated compression screw internal fixation in the treatment of femoral neck fracture in children.

**Methods:**

We retrospectively reviewed the data of ten children with femoral neck fractures treated by robot-assisted internal fixation from January 2020 to June 2021. The clinical and radiological characteristics, operation duration, and fluoroscopy frequency of robot-assisted screws placement together with the complications and function were evaluated. At the 12-month follow-up, the hip joint function was evaluated using the Ratliff classification.

**Results:**

Ten children, six boys and four girls, aged 4–14 years were included. There were eight type II and three type III femoral neck fractures using the Delbet classification. In the process of robot-assisted internal fixation, the median of fluoroscopy frequency was 22 times and the median of operation duration was 47 min. The median of screw parallelism was 1.33° and 0.66° on the anteroposterior and lateral x-ray films, and the median of screw distribution was 41.86% and 44.93% on the anteroposterior and lateral x-ray films, respectively. At the 12-month follow-up, there were two cases of femoral head necrosis, and fracture healing was achieved in all patients, of which eight fractures were excellent and three were good by the Ratliff function classification.

**Discussion:**

The application of robot-assisted cannulated compression screw internal fixation could help us achieve more safe and accurate screw placement, as well as a good treatment effect for children’s femoral neck fractures.

**Level of Evidence:**

Level IV. retrospective case series.

## Introduction

Femoral neck fracture in children accounts for approximately 1% of the total number of fractures in children (1–3). This low incidence is probably owing to the severe trauma, such as that caused by a traffic accident injury or falling injury, needed to fracture the tough, dense bone in children with its thick and strong periosteum (4–6). Owing to the presence of the epiphyseal plate in the proximal femur of children and the vulnerable blood supply to the femoral head, effective treatment of this potentially hazardous fracture is the key to a successful outcome. Ineffective treatment frequently leads to various complications and adverse consequences (1, 7).

Closed reduction and internal fixation with cannulated screws is the main surgical method for the treatment of femoral neck fractures in adults. Some studies have confirmed that exact screw placement enables a biomechanically stable fixation and reduces the risk for fracture nonunion (8–10). This technique is gradually being applied to treat femoral neck fractures in children. Owing to the small diameter of the femoral neck in children, satisfactory screw placement is very difficult. An experienced orthopaedic surgeon is required to perform the operation by hand under fluoroscopy, and even adjust the pin insertion direction repeatedly, which may reduce proximal femur stability. Moreover, repeated x-ray exposure increases radioactive damage to patients and medical personnel, resulting in iatrogenic damage (11–13).

In recent years, robot-assisted internal fixation technology has been ever more widely used in the adult femoral neck fracture operation (14–16). Studies have shown that the use of a robot can determine the ideal placement position of the screws according to the anteroposterior (AP) and lateral images after the reduction, and subsequently provide the placement channels of guide pins with its mechanical arm according to the planned route (17, 18). Surgery simulation experiments showed that the robot-assisted internal fixation can improve surgical accuracy, reduce the number of drilling attempts and intraoperative radioactive damage, and does not increase the operation time (19). However, there are few reports on robot-assisted cannulated compression screw internal fixation for femoral neck fracture in children. Our study summarizes the clinical data of ten children who were treated with robot-assisted internal fixation in our hospital from January 2020 to June 2021.

## Materials and methods

### General clinical data

We retrospectively reviewed the data of ten children (11 femurs) with femoral neck fractures in our hospital from January 2020 to June 2021. Inclusion criteria were: (1) femoral neck fracture; (2) treatment with cannulated screws using the robot navigation system; (3) aged under 18 years. Exclusion criteria were: (1) pathological fracture of the femoral neck; (2) a history of hip fracture on the affected side; (3) follow-up data were incomplete or the follow-up period was <12 months. A total of ten patients were treated with robot-assisted internal fixation, including six males and four females, aged 4–14 years. One patient had a bilateral femoral neck fracture, while the others were unilateral. Among the causes of injury, there were four cases of falling injury, five of traffic accident injury, and one of sport injury. Multiple injuries included one case of perineal anus avulsion injury and urethral rupture, five of pulmonary contusion, two of liver and spleen contusion, and one of pancreatic injury. All patients had skin and soft-tissue injuries. According to the Delbet classification (20), there were eight cases of type II and three cases of type III femoral neck fracture in our group. Basic information about the patients were shown in Table 1.

**Table 1:** Basic information of 10 children with femoral neck fracture.

Patient	Injury side	Sex	Age (yr + mo)	The time from injury to surgery (d)	Delbet classification	Causes
1	L	M	5 + 10	6	III	Falling injury
	R				III	
2	R	F	6 + 3	3	II	Falling injury
3	R	M	4 + 5	7	II	Car accident
4	L	M	5 + 7	6	II	Car accident
5	L	M	6 + 6	5	II	Car accident
6	L	F	12 + 10	5	I	Falling injury
7	R	F	8 + 10	5	II	Sport injury
8	L	M	14 + 2	2	III	Car accident
9	R	M	14 + 7	4	II	Falling injury
10	R	F	9 + 11	5	II	Car accident

### Surgical procedure

**Figure 1:**
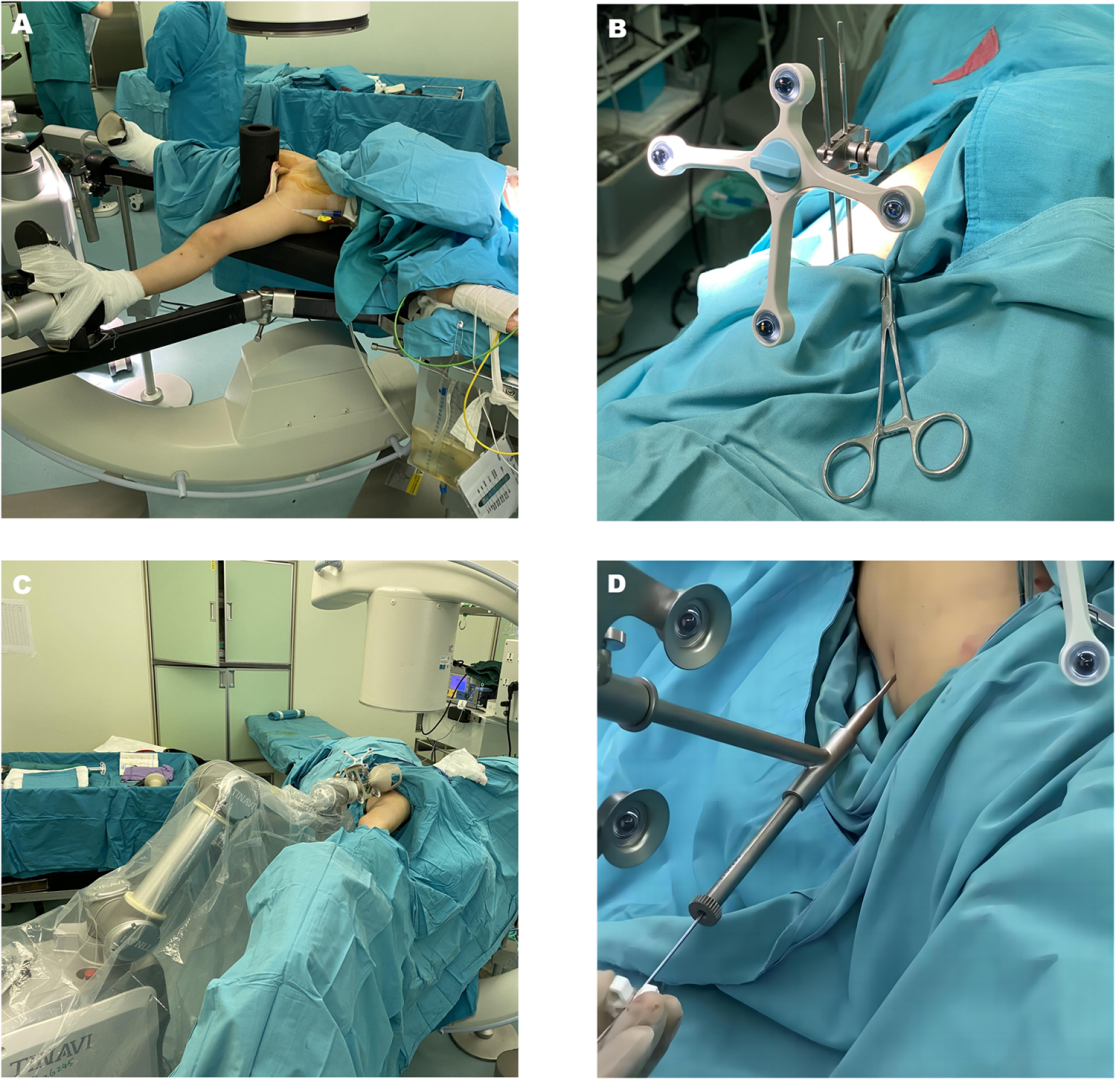
The key surgical steps of the robot-assisted surgery. (**A**) The patient was placed on a traction bed; (**B**) The patient tracking device was fixed on the anterior superior iliac spine of the affected side. (**C**) Obtain the intraoperative fluoroscopy image containing the robot positioning marking point; (**D**) Placed a guide pin near the skin through a sleeve on mechanical arm.

Following general anaesthesia, the patient was placed on a traction bed (Figure 1). Closed reduction was performed for all femoral neck fractures. Following satisfactory reduction, three cannulated screws in an inverted triangular distribution were placed using a bi-planar navigation robot, TiRobot (TINAVI Medical Technology Company, Beijing, China), which consists of a multi-degree-of-freedom mechanical arm, an optical tracking device, a workstation for surgical planning and the controlling system.

**Figure 2:**
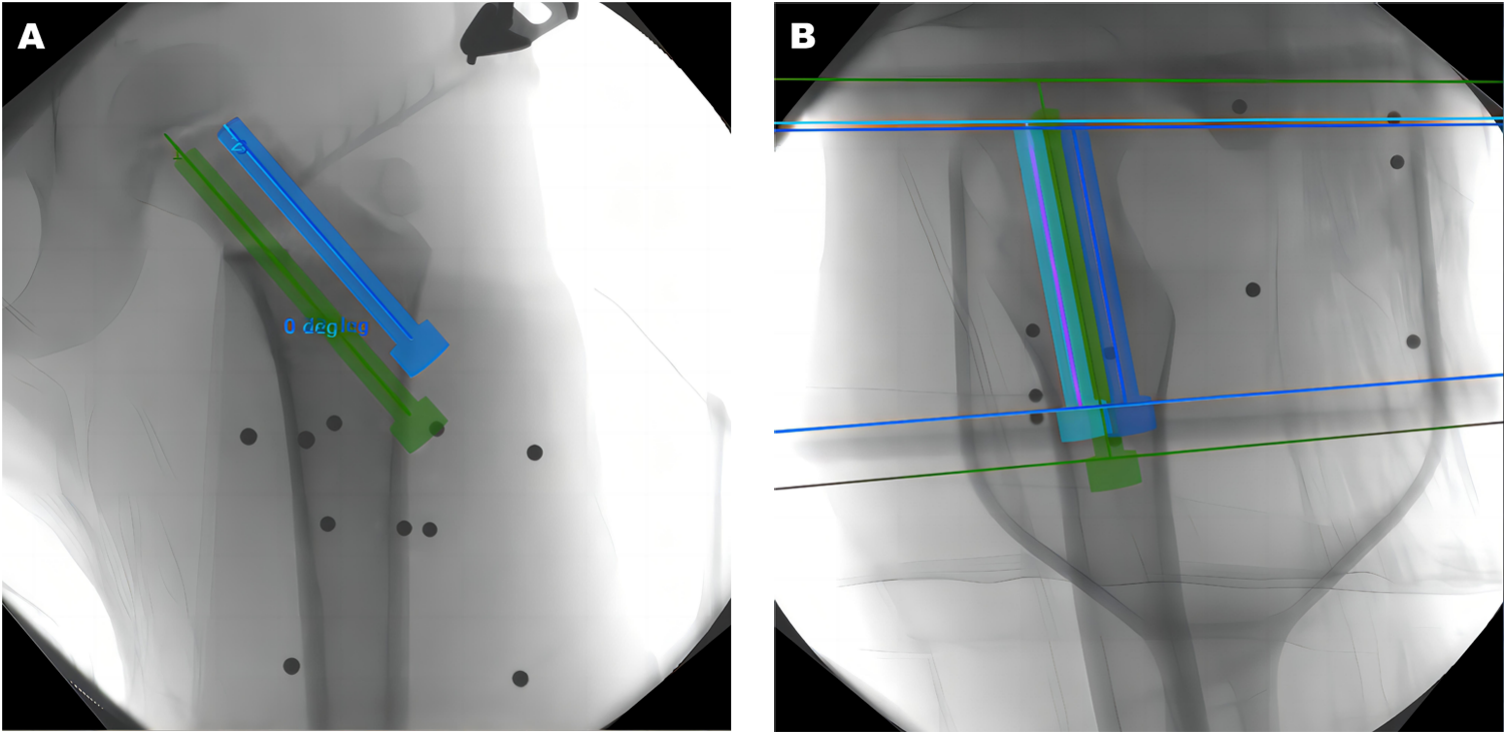
A 5-year-old boy with left femoral neck fracture. All ten positioning marking points should be clearly displayed and transmitted to the workstation. Path planning of screw placement as shown in the AP (**A**) and lateral (**B**) images.

**Figure 3:**
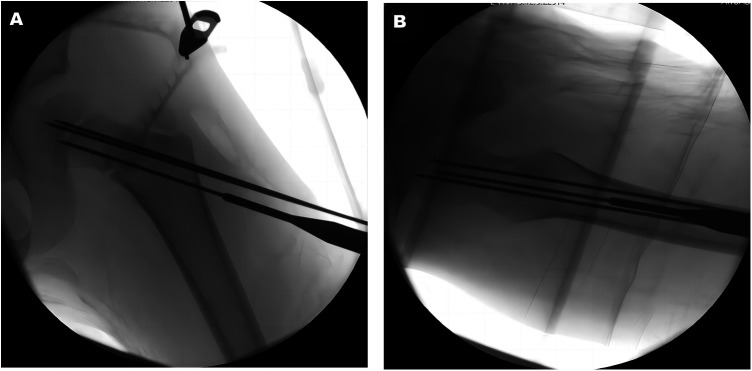
Three guide pins were drilled into the bone passage in sequence through the sleeve under fluoroscopy monitoring as shown in the AP (**A**) and lateral (**B**) images.

**Figure 4:**
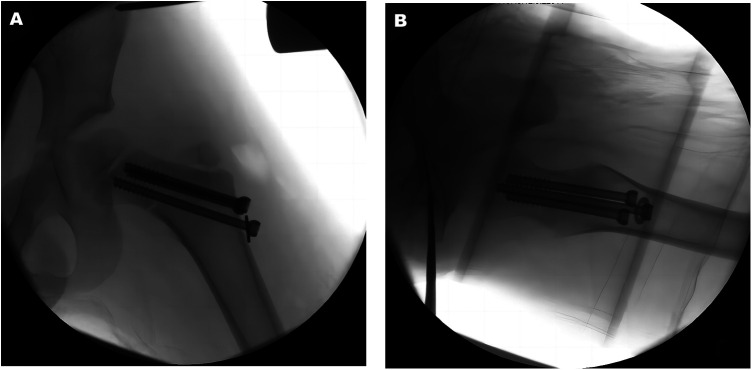
Three cannulated compression screws were inserted along the guide pins, and the guide pins are removed as shown in the AP (**A**) and lateral (**B**) images.

The patient tracking device was fixed on the anterior superior iliac spine of the affected side. The C-arm is used to obtain the intraoperative fluoroscopy image containing the robot positioning marking points. All ten positioning marking points should be clearly displayed in the images and transmitted to the workstation for registration calculation. The operator designed the locations of the three screws in the software according to the patient's fracture status, and inputted the screw diameter, designed the screw insertion direction and depth according to whether the screw passes through the epiphyseal plate. The three screws should be parallel to each other in an inverted triangle (Figure 2). Once the first screw was selected, the system ran the mechanical arm to the target position according to the planning path. The surgeon placed a guide pin near the skin through a sleeve; the guide pin should not penetrate the skin; an intraoperative fluoroscopy was performed to confirm that the direction of the guide pin was consistent with the planned direction. Subsequently, the surgeon marked the remaining two screw insertion points in the same way. On the basis of the location of the three screws' insertion points, a uniform incision was selected that allowed the three screws to be drilled at the same time. Once the surgical approach was ready, three guide pins were drilled into the bone passage in sequence through the sleeve under fluoroscopy monitoring (Figure 3). Finally, after confirming the good position and depth of the cannulated screws, the guide pins were removed and the surgical incision was sutured (Figure 4).

### Postoperative treatment and follow-up

The affected limb was immobilized with a single-leg spica cast or brace after surgery. Intravenous antibiotics were routinely used for 48 h during perioperative period. If combined with other organ injuries, the length of antibiotic administration depends on the damage to other organs. The day after surgery, patients were encouraged to perform non-weight-bearing training of the lower extremities, such as quadriceps contraction. Four weeks after surgery, the brace was removed and partial non-weight-bearing exercise such as adduction and abduction of the hip was performed. The AP and lateral x-ray films of the femoral neck were repeated once a month to observe the fracture recovery. Six months after surgery, weight-bearing exercise was gradually performed when the x-ray showed fracture healing.

### Imaging and functional evaluation

**Figure 5:**
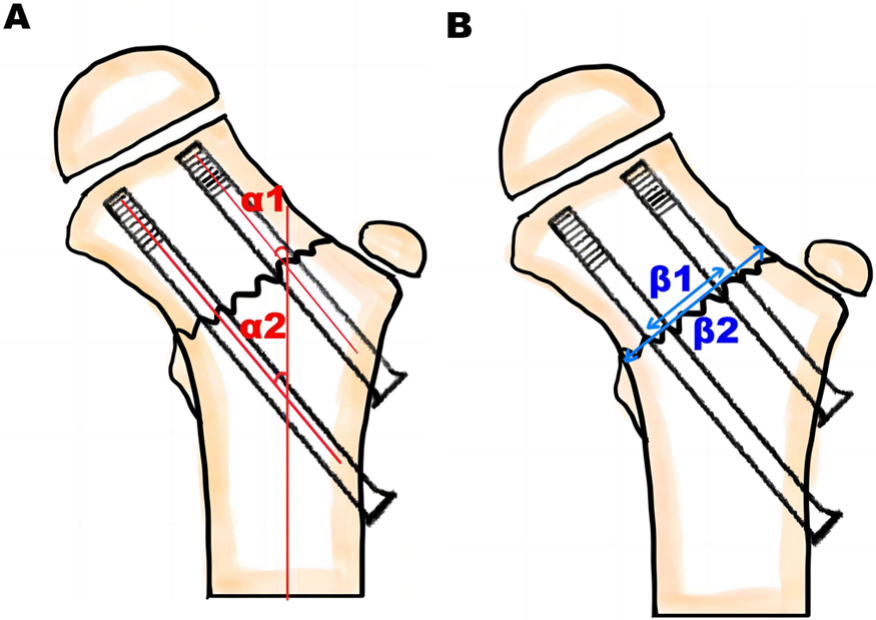
Evaluate the screw parallelism and screw distribution. (**A**) The shaft screw angle (*α*) was defined as the angle between the femoral shaft axis and the longitudinal axis of each screw. (**B**) The screw distribution was defined as the relative coverage of the neck width, which was calculated as the product of the distance between the borders of the outer screws at the fracture line divided by the neck width at the fracture level.

Eight cases of femoral neck fracture were reduced by abduction and internal rotation under continuous traction, one was reduced by abduction and external rotation under continuous traction, and in two hip flexion and abduction were reduced without traction. During the operation, the reduction effect was observed by C-arm of the femoral neck in the AP and lateral positions. The fracture reduction quality was evaluated using the Haidukewych score (21). AP and lateral radiographs of the hip joint were obtained postoperatively to evaluate the parallelism and screw distribution. The shaft screw angle (*α*) was defined as the angle between the femoral shaft axis and the longitudinal axis of each screw (Figure 5). Screw parallelism was calculated as the mean difference between the shaft screw angle, screw parallelism = (|*α*1–*α*2| + |*α*1–*α*3| + |*α*2–*α*3|)/3. On the basis of the standard postoperative AP and lateral x-ray images, if the screw parallelism was <10°, the screws placement was considered to be parallel and the position to conform to the ideal position (22). The screw distribution was defined as the relative coverage of the neck width, which was calculated as the product of the distance between the borders of the outer screws at the fracture line divided by the neck width at the fracture level. The screw distribution = *β*1/*β*2 × 100%, where *β*1 is the distance between the superior border of the proximal screw and the inferior border of the most distal screw and *β*2 is the width of the femoral neck at the fracture level (Figure 5). If the screw distribution was ≥20% in the AP x-ray and ≥25% in the lateral x-ray, this was considered to fit the ideal position (22, 23).

Following surgery, the fluoroscopy frequency and the operation duration were calculated according to the fluoroscopy records automatically saved in the C-arm system (Siemens, Germany). This started with the collection of the femoral neck images after the reduction, and ended with the satisfactory fluoroscopy position after the placement of the three screws. Finally, the mean fluoroscopy frequency and operation duration of the 11 cases were recorded. Complications, including premature epiphyseal closure, coxa varus deformity, and femoral head necrosis, were recorded at the 12-month follow-up. Avascular necrosis (AVN) of the femoral head was classified and the hip joint function was evaluated from the four aspects of pain, hip joint mobility limitation, exercise situation, and femoral neck x-ray appearance according to the Ratliff classification (2). The treatment effect was classified into three grades: excellent, good, and poor.

### Statistical analysis

Statistical software IBM SPSS 26.0 (International Business Machines Corporation, New York, United States) was used for statistical analysis. The quantitative data included the operation time, the fluoroscopy frequency, and the angle of screw parallelism and distribution on the AP and lateral x-ray films, respectively. The measurement data were tested by S-W normal distribution test. If the data conform to the normal distribution, the mean and standard deviation are calculated, and if not, the median and interquartile range are used.

## Results

Ten children, six boys and four girls, aged 4–14 years were included. There were four cases of falling injury, five of traffic accident injury, and one of sport injury. According to the Delbet classification of femoral neck fracture, there were eight type II cases and three type III cases. According to the Haidukewych reduction standard, ten cases were excellent and one was good. During the process of the surgery, the median of fluoroscopy frequency was 22 times and the median of operation duration was 47 min. The median of screw parallelism was 1.33° and 0.66° on the AP and lateral x-ray films, and the median of screw distribution was 41.86% and 44.93% on the AP and lateral films, respectively. The intraoperative statistics of ten children are shown in Table 2.

**Table 2:** The intraoperative statistics of ten children with femoral neck fractures treated by robot assisted internal fixation.

	Median	Interquartile range
Fluoroscopy frequency (times)	22.00	18.00
Operation duration (min)	27.00	25
Screw parallelism, AP^a^ (°)	1.33	0.66
Screw parallelism, lateral (°)	0.67	0.66
Screw distribution, AP^a^ (%)	41.86	6.61
Screw distribution, lateral (%)	44.93	11.78

^a^ AP, anteroposterior.

During the follow-up period, there was no infection, loosening of internal fixation, or fracture displacement. There were two cases of femoral head necrosis, including one Ratliff type I and one type II. At the 12-month follow-up, fracture healing was achieved in all patients, of which eight were excellent and three were good according to the Ratliff function classification.

## Discussion

Femoral neck fractures in children are often caused by high-energy injuries and are severely displaced, often with serious consequences. Satisfactory reduction and reliable internal fixation are the key to treatment (24). Therefore, we tried to use robot-assisted internal fixation to treat femoral neck fractures in children, in order to achieve good treatment results. Femoral neck fractures in children are uncommon because of the strong structure of their proximal femoral bone, thus significant force is required for breakage, whereas minor trauma is the main etiological factor in adults (6, 25). Our data confirmed the etiological factors, because all the fractures were caused by high-energy trauma. Among the causes of injury, there were four cases of high-level falling injury, five of traffic accident injury, and one of sport injury. Owing to the vascular system of a child is terminal, and blood vessels cannot cross the open physis, the blood supply to the femoral head is critical and can be readily disrupted by a hip fracture in children (25). Therefore, the blood supply to the femoral head is critical and can be readily disrupted by a hip fracture in children. The risk of AVN depends on several factors, including the degree of initial displacement, fracture type, time from injury to surgery, surgical protocol, and fixation method. The most important factor is likely to be the severity of vascular compromise sustained at the time of trauma. Therefore, AVN is the most common and damaging complication in paediatric femur neck fractures (26). Ineffective treatment frequently leads to various complications and adverse consequences. In our study, there were two cases of femoral head necrosis, including one Ratliff type I and one type II. The possible causes of AVN after surgery in our cases may be due to the high-energy injury, which caused severe displacement of the femoral neck fracture and obvious destruction of blood supply to the femoral head. At the same time, the early stage of treatment was mainly to maintain children's vital signs, and there was no early reduction and fixation or capsulotomy decompression of femoral neck fracture. In our cases, the rate of AVN was 18.18% which was slightly less as compared with previous studies. Yeranosian et al. (27) reported that a mean of 22% of cases developed AVN, with the incidence decreasing from about 40% in Delbet type I fractures to around 5% in type IV. This may be because AVN can appear two years or more after injury (27). The low incidence of AVN in our study may be due to the short follow-up time.

Robot-assisted cannulated compression screw fixation has been proven to be an effective surgical technique in recent years and has been widely applied in spinal and adult orthopaedics (19, 28–30). It enables surgeons to perform detailed surgical planning and precise surgical manipulations, reducing the fatigue of the doctor from long-time device holding and avoiding problems such as changes in the guide pin placement direction (22, 31, 32). The ideal distribution of the three screws in the femoral neck fracture is parallel and in an inverted triangle, and dispersed as close as possible to the femoral neck cortex to enhance the stability of fracture fixation and promote fracture healing (10, 13, 33). In the process of using the orthopaedic surgical robot for screw insertion, the surgeon can drill the guide pin accurately through the insertion channel provided by the sleeve on the mechanical arm under the real-time monitoring to make the post-operative screw placement more parallel (23). Wang et al. (18) compared the fluoroscopy frequency and operation duration of robot assisted surgery and traditional surgery in the treatment of adult femoral neck fractures, and the data of robot group was lower than that of traditional group. He et al. (14) compared the parallelism and distribution of two different surgical methods, The postoperative screw parallelism and distribution in the robot assisted group were significantly better than those in the traditional group, there was significant difference between the two groups. At the same time, there is an epiphyseal plate in the proximal femur of children, and internal fixation through this has the risk for premature epiphyseal closure. Although we consider stable reduction more important than epiphyseal closure during surgical reduction, in children aged younger than 10 years, epiphyseal penetration greatly increased the possibility of developing premature epiphyseal closure (1). Robot-assisted screw fixation surgery can control the placement depth and direction during the surgical placement process to avoid the screws passing through the epiphyseal plate.

Because the femoral neck of children is thinner than that of adult and the interference of the fascial tissue may cause a small positional change of the sleeve, the surgical approach must be fully exposed, and after the sleeve contacts the bone surface, fluoroscopy is performed to again ensure that the sleeve path is consistent with the planned path. Surgeons mostly choose 4.5 mm diameter screws to treat femoral neck fractures in children and the corresponding guide pin diameter is 1.4 mm. When the guide pin passes through the bone cortex, it can readily deform and affect the pin direction. Therefore, the guide pin should pass through the bone cortex at high speed without applying forward thrust to prevent it deforming and changing its path. Among the ten children we treated, one patient received continuous abduction and external rotation traction to achieve a satisfactory reduction effect. However, this posture made it very difficult to insert the three screws. To solve the difficulty of screw placement, a guide pin in the centre of the femoral neck was designed and fix the fracture by robot-assisted temporarily. Subsequently, the hip joint was placed in the neutral position, the three screw paths of the femoral neck fracture were redesigned and the guide pins and compression screws were placed to reduce the impact of the body position on the screw placement.

Robot assisted surgery does not require the collection of three-dimensional image information of the femoral neck, but only of the two-dimensional images of the AP and lateral positions of the femoral neck. In addition, in the screw placement process, with the help of the robot arm, the screws are accurately placed, avoiding multiple adjustments, thereby reducing the damage of multiple radiation exposures to the patient and medical personnel. Although robot assisted screw placement technology can help surgeons to accurately place screws, the path planning of screw placement still relies on the experience of surgeons. The reduction quality and reasonable path planning before pin placement are important factors in determining fracture healing and AVN of the femoral head. Satisfactory surgical results can only be achieved through satisfactory fracture reduction, reasonable screw distribution, and accurate screw placement.

Owing to the short time required when using robot-assisted internal fixation in the treatment of femoral neck fractures in children, and because the incidence of this fracture type is low, we have, to date, not compared it with the traditional non-robot assisted compression screw internal fixation. Our next study is to compare the differences between robotic and tradition because the incidence of this fracture type is low, we have, to date, not compared it with the traditional non-robot assisted compression screw internal fixation al surgical treatment of children's femoral neck fractures, and further evaluate the effectiveness of robotic technology in treating such fractures in children.

## Conclusion

The application of robot-assisted cannulated compression screw internal fixation could help us achieve more safe and accurate screw placement, as well as a good treatment effect for children's femoral neck fractures. In the future, more high-quality randomized controlled trials are needed to verify this fixation technique.

## Data Availability

The original contributions presented in the study are included in the article/Supplementary Materials, further inquiries can be directed to the corresponding author/s.
